# Distributed Extended Kalman Filtering Based Techniques for 3-D UAV Jamming Localization

**DOI:** 10.3390/s20226405

**Published:** 2020-11-10

**Authors:** Waleed Aldosari, Muhammad Moinuddin, Abdulah Jeza Aljohani, Ubaid M. Al-Saggaf

**Affiliations:** 1 Center of Excellence in Intelligent Engineering Systems, King Abdulaziz University, Jeddah 21589, Saudi Arabia; waldosari@kau.edu.sa (W.A.); mmsansari@kau.edu.sa (M.M.); usaggaf@kau.edu.sa (U.M.A.-S.); 2 Department of Electrical and Computer Engineering, King Abdulaziz University, Jeddah 21589, Saudi Arabia

**Keywords:** jamming attacks, Unmanned Aerial Vehicle (UAV), localization, Extended Kalman Filter (EKF)

## Abstract

Wireless networks are vulnerable to jamming attacks. Jamming in wireless communication becomes a major research problem due to ease in Unmanned Aerial Vehicle (UAV) launching and blocking of communication channels. Jamming is a subset of Denial of Service Attack (DoS) and an intentional interference where the malicious node disrupts the wireless communication by increasing the noise at the receiver node through transmission interference signal towards the target channel. In this work, the considered jammer is a UAV hovering around the target area to block the communication channel between two transceivers. We proposed a three-dimensional (3-D) UAV jamming localization scheme to track and detect the jammer position at each time step by employing a single boundary node observer. For this purpose, we developed two distributed Extended Kalman Filter (EKF) based schemes: (1) the Distributed EKF (DEKF) scheme using the information of the received power from the jammer at a single nearby boundary node only and (2) Distance Ratio aided Distributed EKF (DEKF-DR) based scheme utilizing an edge node in addition to a single boundary node. Extensive simulations are conducted in order to evaluate the performance of the proposed distributed algorithms for a 3-D trajectory and compared with that of the conventional Centralized EKF (EKF-Centr) based method (which is also modified for the 3-D scenario). The results show the clear supremacy of the proposed distributed algorithms with much lesser complexity in contrast to the conventional EKF-Centr technique.

## 1. Introduction

Unmanned Aerial Vehicles (UAVs) are an emerging technology designed to flight with no human pilot. The UAV use was initially motivated by military applications, including reconnaissance, surveillance, and tracking [1,2]. This is due to the fact that the UAVs can be readily equipped with sensors, cameras, radars, and more technologies for military purposes [3,4]. Recently, the use of UAV has unprecedentedly increased including wide range of applications, such as, public safety, police, transportation, package delivery, and environmental monitoring. UAVs offer crucial help in rescue and recovery for disaster relief operations, when public communication networks get crippled [5], since they can form a salable and dynamic networks [6]. The UAV ability of hovering over a targeted area is desirable in many applications, for example, a UAV can help in localization when the Global position System (GPS) unavailable or inaccurate [7,8].

Jamming attacks in wireless communication can be classified into two main levels [9,10]. First, the elementary level that includes constant, deceptive, and random jammers. The constant jammer simply transmits a random bit continually to make the available channel busy [10]. Hence, any node within the jammer transmission range will be unable to access the channel and keep sensing until its battery depleted [9]. While the deceptive jammer, as the name suggested, tries to mimic the original transmission by sending regular packets towards the target node. In this case, all of the nodes located around the jammer will switch to receive mode and process the received corrupted data. The random jammer switches to active mode and transmitting jamming signals towards the target for a certain time and then return to sleep mode [11,12]. Second, the advanced level that might include the barge and the spot jammers. Here, the jammers sense all available channels; once it detects the channel used by legitimate one, it immediately transmits its signal to jam it. The jammers change their frequencies from carrier to another aiming to block all sub-channels [13]. Detecting the UAV jamming location is the first step towards preventing such an attack. By finding the UAV location, any appropriate action can be taken against the enemy UAV; e.g., physically destroying it or jam the Jammer by another jamming source [14,15].

Tracking and localization jammers in wireless communication networks are still challenging topics. The used algorithms can be categorized into two main groups: range-free and range-based schemes [16]. The accuracy of the range-free techniques is based on nodes locations and the change of network topology. In this context, the Centroid Localization (CL) algorithm [17] and Weighted Centroid (WCL) algorithm [18] are examples of range-free techniques. They are both sensitive to node locations and the number of nodes deployed. The location accuracy increases when the number of nodes affected by the jamming signal increases [18,19]. Therefore, the range free method is inaccurate when the affected nodes are fewer and are located closer to each other. However, the range-based method utilizes the so-called Jammer Received Signal Strength (JRSS) in order to detect and predict jammers’ physical locations. This method tries to estimate the JRSS of the original signal, hence more reliable results can be achieved when compared to its range-free counterpart. Centralized Extended Kalman Filtering (EKF-Centr) was proposed in [20], where the algorithm is based on the received jammer’s power from the boundary nodes at each time step. It was found [20,21] that the tracking efficiency increases when the number of boundary nodes increases.

A method for detecting jammer’s location utilizing the Packet Delivery Ratio (PDR) rate at each node was reported in [22]. In [23], the adaptive RSSI filtering technique is employed to improve the measured RSSI signal degraded due to multipath effects with the aim to enhance the localization accuracy and to reduce the computational complexity of the tracking system. The RSSI based approaches that Kalman filtering to estimate the target position are presented in [24,25]. They considered that, during jamming attacks, both the Signal to Noise Ratio (SNR) and PDR decrease as the amount of noise increases. Therefore, any node that has a lower PDR than expected is considered as a near jammer node and the gradient descent technique is employed in order to track the jamming source [22]. Wide-band jammer localization method introduced in [26], where a combination of Difference of Arrivals (DOA), Time Difference of Arrivals (TDOA), and EKF used. More explicitly, the DOA was utilized in order to provide the EKF with an accurate initial position, while the TDOA helped the EKF for fast converge. The efficiency of this method depends on the number nodes used for localization and tracking processes [26].

In summary, the aforementioned existing solutions have one of several drawbacks, such as (i) higher computationally complex due to heavy computational requirements for DOA/TDOA estimation [26], (ii) sensitive to node locations and the number of nodes deployed [18,19], (iii) require a large computations for centralized processing and dependency on large number of boundary nodes [20], and (iv) highly sensitive to noise power (or SNR) [22]. Against these techniques, we proposed two novels distributed Extended Kalman Filter (EKF) based schemes:

- Distributed EKF (DEKF) scheme while using information of the received power from the jammer at a single boundary node only.
- Distance Ratio aided Distributed EKF (DEKF-DR) based scheme utilizing an edge node in addition to a single boundary node.

In general, these two methods require less computation due to distributed processing. Among these methods, DEKF-DR is the most promising, as it only uses a single node and its one neighbor to estimate received powers while offering the best accuracy when compared to the DEKF and the EKF-Centr (which is also modified for the 3-D scenario) techniques.

The paper is organized, as follows: following the introduction, the system model is presented in Section 2. Section 3 introduces the concept of Distance Ratio. In Section 4, an overview of the standard Extended Kalman Filter is provided and the proposed distributed Extended Kalman Filter for 3-D Jammer’s Localization are developed. Section 5 provides extension of the existing centralized EKF-aided jammers Localization scheme to 3-D scenario. The results and discussion are provided in Section 6. Finally, concluding remarks are given in Section 7.

## 2. System Model

**Figure 1:**
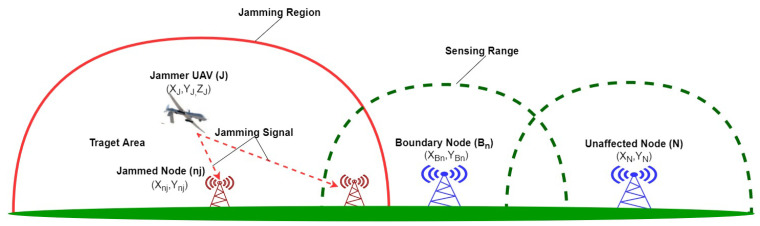
Jammer Unmanned Aerial Vehicles (UAV) targeted two base stations on the ground where the blue base station represents unaffected nodes and the red base stations are the jammed nodes. The dot green line depicts the sensing range and the solid red line the jamming region.

**Figure 2:**
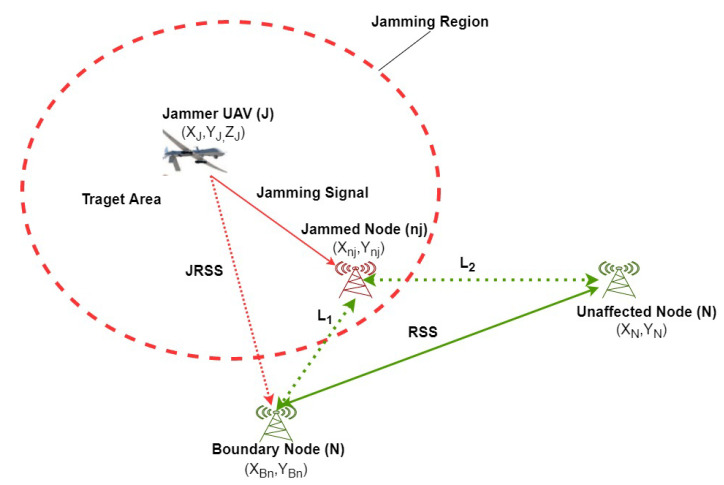
Representation of the link quality and JRSS while jammer is moving. The solid arrow describes the jamming signal and the dotted red line is the JRSS received by the base station. The link between the nodes depicted by the dot green line and the power received by the boundary node from its neighbor represented as a solid green line.

We consider a jammer UAV (J) flying above the target area and emitting its jamming signal towards base stations on the ground. There are two base stations located outside the jamming region that may transmit and receive data from each other, as shown in Figure 1. Without loss of generality, we consider that the jammer UAV is moving in a three-dimensional Cartesian coordinates system with variable speed and constant acceleration. Constant acceleration is used in many works such as in [4] and non-acceleration model [27]. Non-acceleration models are commonly used in monocular SLAM systems [27]. The base stations are at fixed positions in the XY-plane, as shown in Figure 2. During a jamming attack, the network topology may change and nodes will be affected based on their locations and jammer’s transmission power. Note that, the key parameters are defined in Table 1.

**Figure 3:**
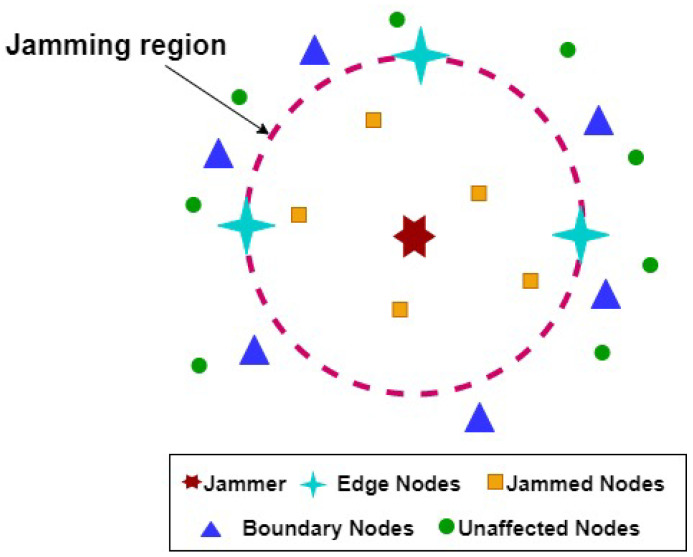
Nodes classification during jamming attack.

Based on jamming signal and noise powers at the received signal by sensors, the nodes are classified into three different types [28], as shown in Figure 3. When jamming signal attacks nodes, the SNR decreases which result in increased Bit Error Rate (BER) and decreased Packet Delivery Ratio (PDR). Thus, the system fails to decode the signal properly and it requests the re-transmission of signals. Packets received with SNR below the system threshold value $\left(SNR<\gamma \right)$ are considered invalid data and require re-transmission of data. The sensors located inside the jamming region are called Jammed Nodes, $N_{J}$, and have SNR less the than system threshold value (γ). Hence, they are completely isolated from the network and cannot send or receive data from their neighbors. On the other hand, the nodes located near the jamming region and have SNR larger than the system threshold are known as the Boundary Nodes, *B*. These nodes lost their links with some of their neighbors. However, they might still have active links with unaffected sensors and can communicate with them. Lastly, the nodes outside the attacked area with SNR larger than the system threshold value are called Unaffected Nodes *N*.

## 3. Concept of Distance Ratio (β)

**Figure 4:**
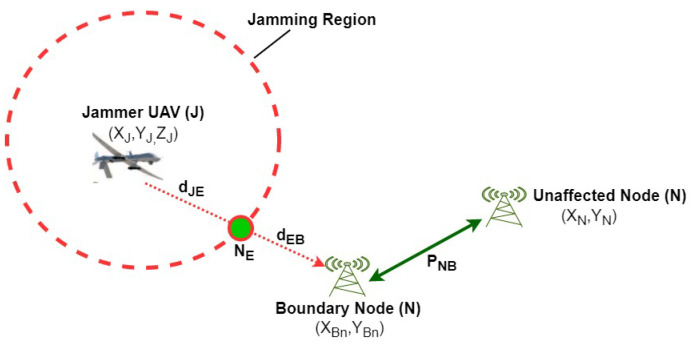
Illustration of distance ratio. The two base stations positioned outside the jamming range wherever the green circle depicts the edge node and placed at the jamming region.

In this section, we described the concept of distance ratio which was introduced in [29]. Moreover, we extended the distance ratio concept to three-dimensional space $\left(x,y,z\right)$. This concept will be later utilized in order to develop the EKF algorithm in Section 4. Note that, Table 1 defines the main parameters. The distance ratio concept is based on signal to noise ratio at the edge node $N_{E}$, jamming power received by boundary node *B* and Received Signal Strength (RSS) from *B*, as shown in Figure 4. Here, $d_{JB}$ and $d_{JE}$ are the distances from the jammer to the boundary node and from the jammer to the edge node, respectively. Thus, we can express $d_{JB}$ and $d_{JE}$, respectively, as:

(1) $$d_{JB}=\sqrt{{\left(x_{B}-x_{J}\right)}^{2}+{\left(y_{B}-y_{J}\right)}^{2}+{\left(z_{B}-z_{J}\right)}^{2}}$$

and

(2) $$d_{JE}=\sqrt{{\left(x_{E}-x_{J}\right)}^{2}+{\left(y_{E}-y_{J}\right)}^{2}+{\left(z_{E}-z_{J}\right)}^{2}}$$

The SNR decreases when the jammer moves towards the target node until it drops to below an acceptable threshold value. This results in a jammed node and communication channel being completely blocked. Therefore, the node has SNR approximately equal to the system threshold value $\left(SNR\approx \gamma \right)$ located on the jamming edge. We use this observation in order to estimate the unknown distances between the jammer and edge node. The jamming power received by the boundary node is following the Log-distance shadowing model where it an extension of the Friis equation and it is proportional inverse to the distance, as follows:

(3) $$P_{JB}=P_{t}+k-10n\log_{10}d_{JB}+X_{\sigma }$$

where $P_{JB}$ is the jamming power received at distance *d*, and $P_{t}$ is the jammer’s transmission power, the path loss exponent *n*, depending on the environment and it is variant from physical environment to other. In this paper we assumed it equal to 2 for free space environment, or Line of Sight (LoS). The Gaussian noise with zero mean denoted by $X_{\sigma }$. *k* is constant and depend on the antenna characteristics. $d_{JB}$ is the distance between jammer and the boundary node.

Similarly, the jamming power received by the edge node (represented by $P_{JE}$) can be expressed as:

(4) $$P_{JE}=P_{t}+k-10n\log_{10}d_{JE}+X_{\sigma }$$

Next, we define the distance ratio (β) as the ratio of $d_{JE}$ to $d_{JB}$, that is:

(5) $$\beta =\frac{d_{JE}}{d_{JB}}$$

Now, using the geometry that is shown in Figure 4, we conclude that the distances $d_{JE}$, $d_{JB}$, and $d_{EB}$ are related as:

(6) $$d_{JB}=d_{JE}+d_{EB}$$

Thus, we can also relate $d_{EB}$ and $d_{JB}$ while using the following:

(7) $$\left(1-\beta \right)=\frac{d_{EB}}{d_{JB}}$$

As a result, we can relate the power terms $P_{JB}$ and $P_{JB}$ with the aid of distance ratio to obtain:

(8) $$P_{JE}=P_{JB}-10n\log_{10}\left(\beta \right)$$

Similarly, it can be shown that $P_{EB}$ and $P_{JB}$ can be related as:

(9) $$P_{EB}=P_{JB}-10n\log_{10}\left(1-\beta \right)$$

Later, in Section 3, we will explain how the above equations can help us in the development of a distributed Extended Kalman Filter (EKF) algorithm for the jammer’s 3D location estimation.

## 4. Proposed Distributed Extended Kalman Filter Based 3D Jammer’s Localization Schemes

The Extended Kalman Filtering is an extension of Linear Kalman Filter (LKF), which is capable to take into account the nonlinearity of the system model. More specifically, the EKF employs the first-order linearization of the nonlinear system in a recursive fashion to find the estimates current mean and the covariance of the state vector [30].

Consider the following non-linear state transition model:

(10) $$x_{k}=f\left(x_{k-1},u_{k}\right)+w_{k}$$

where $x_{k}$ represents the state vector, $u_{k}$ is the input vector, f represents the non-linear state transition function, and $w_{k}$ is a zero mean Gaussian process noise with a covariance matrix Q, i.e., $w_{k}∼N\left(0,Q\right)$. Now, the measurement vector $z_{k}$ can be expressed as:

(11) $$z_{k}=h\left(x_{k},u_{k}\right)+v_{k}$$

where $v_{k}$ is a zero mean Gaussian measurement noise with a covariance matrix R, i.e., $v_{k}∼N\left(0,R\right)$. Here, h represents non-linear function for the measurement process. The EKF estimates the current position in two main steps, prediction and update state [31]. Next, we can define the following Jacobian matrices as:

(12) $$A_{k}=\frac{\partial f(x_{k-1},u_{k})}{\partial x_{k-1}}{|}_{{\hat{x}}_{k-1|k-1}}\;\;\;and\;\;\;H_{k}=\frac{\partial h(x_{k},u_{k})}{\partial x_{k}}{|}_{{\hat{x}}_{k|k-1}}$$

the conventional EKF algorithm can be summarized, as follows:

(13) $${\hat{x}}_{k|k-1}=f\left({\hat{x}}_{k-1|k-1},u_{k}\right)$$

(14) $$P_{k|k-1}=A_{k}P_{k-1|k-1}A_{k}^{T}+Q_{k}$$

(15) $$K_{k}=P_{k|k-1}H_{k}^{T}{\left(H_{k}P_{k|k-1}H_{k}^{T}+R_{k}\right)}^{-1}$$

(16) $${\hat{x}}_{k}={\hat{x}}_{k|k-1}+K_{k}\left(z_{k}-h\left({\hat{x}}_{k|k-1}\right)\right)$$

(17) $$P_{k|k}=P_{k|k-1}-K_{k}H_{k}P_{k|k-1}$$

where $K_{k}$ is the Kalman gain and $P_{k|k}$ is the state error covariance matrix estimate at time instant k.

Next, we propose our distributed EKF-based two different methods for the 3-D Jammer’s Localization. These are presented in the ensuing sub-sections.

### 4.1. Distributed Extended Kalman Filter (DEKF) for 3D Localization

In this method, a distributed scenario is considered, where each node employs the standard EKF by using the information of the received power from the jammer at the nearby boundary node of the jamming region, as shown in Figure 3. This scheme is termed as Distributed Extended Kalman Filter (DEKF). In the DEKF approach, every node process the jamming power and estimate the jammer’s location locally without the need to collaborate with another node. Thus, for the jammer’s 3D localization task with the DEKF, the state vector will take the following form:

(18) $$x_{k}={\left[x_{J},y_{J},z_{J},v_{x},v_{y},v_{z},a_{x},a_{y},a_{z}\right]}^{T}$$

The Jammer’s motion can be described using the well-known Kinetic equation model given by:

(19) $$x_{k+1}=x_{k}+v\Delta t+\frac{1}{2}a\Delta t^{2}$$

Thus, the Jacobian matrix $A_{k}$ can be expressed as:

(20) $$A_{k}\overset{\Delta }{=}\frac{\partial f}{\partial x_{k}}{|}_{\left(\hat{x}J,k-1,\hat{y}J,k-1,\hat{z}J,k-1\right)}={\left[\begin{array}{cccccccccc} 1 & 0 & 0 & dt & 0 & 0 & \frac{dt^{2}}{2} & 0 & 0\\ 0 & 1 & 0 & 0 & dt & 0 & 0 & \frac{dt^{2}}{2} & 0 & \\ 0 & 0 & 1 & 0 & 0 & dt & 0 & 0 & \frac{dt^{2}}{2}\\ 0 & 0 & 0 & 1 & 0 & 0 & dt & 0 & 0 & \\ 0 & 0 & 0 & 0 & 1 & 0 & 0 & dt & 0 & \\ 0 & 0 & 0 & 0 & 0 & 1 & 0 & 0 & dt & \\ 0 & 0 & 0 & 0 & 0 & 0 & 1 & 0 & 0 & \\ 0 & 0 & 0 & 0 & 0 & 0 & 0 & 1 & 0 & \\ 0 & 0 & 0 & 0 & 0 & 0 & 0 & 0 & 1 & \end{array}\right]}_{\left(\hat{x}J,k-1,\hat{y}J,k-1,\hat{z}J,k-1\right)}$$

Additionally, the covariance matrix of process noise $Q_{k}$ and the covariance matrix of measurement noise $R_{k}$ can be written, respectively, as:

(21) $$Q_{k}=\left[\begin{array}{cccccccccc} \frac{dt^{4}}{4} & 0 & 0 & \frac{dt^{3}}{3} & 0 & 0 & \frac{dt^{2}}{2} & 0 & 0 & \\ 0 & \frac{dt^{4}}{4} & 0 & 0 & \frac{dt^{3}}{3} & 0 & 0 & \frac{dt^{2}}{2} & 0 & \\ 0 & 0 & \frac{dt^{4}}{4} & 0 & 0 & \frac{dt^{3}}{3} & 0 & 0 & \frac{dt^{2}}{2} & \\ \frac{dt^{3}}{3} & 0 & 0 & \frac{dt^{2}}{2} & 0 & 0 & dt & 0 & 0 & \\ 0 & \frac{dt^{3}}{3} & 0 & 0 & \frac{dt^{2}}{2} & 0 & 0 & dt & 0 & \\ 0 & 0 & \frac{dt^{3}}{3} & 0 & 0 & \frac{dt^{2}}{2} & 0 & 0 & dt & \\ \frac{dt^{2}}{2} & 0 & 0 & dt & 0 & 0 & 1 & 0 & 0 & \\ 0 & \frac{dt^{2}}{2} & 0 & 0 & dt & 0 & 0 & 1 & 0 & \\ 0 & 0 & \frac{dt^{2}}{2} & 0 & 0 & dt & 0 & 0 & 1 & \end{array}\right]$$

(22) $$R_{k}=diag\left(\sigma_{v_{x}}^{2},\sigma_{v_{y}}^{2},\sigma_{v_{z}}^{2},\sigma_{a_{x}}^{2},\sigma_{a_{y}}^{2},\sigma_{a_{z}}^{2},\sigma_{P_{JB}}^{2}\right)$$

Now, assume that every sensor node provides the measurements of jammer’s velocity components $\left(v_{x},v_{y},v_{z}\right)$, acceleration $\left(a_{x},a_{y},a_{z}\right)$ components, and the power received, which are captured by boundary node denoted as $P_{JB}$. Hence, the measurement vector can be set up as:

(23) $$z_{k}={\left[v_{x},v_{y},v_{z},a_{x},a_{y},a_{z},P_{JB}\right]}^{T}$$

Thus, in this scenario, both the observation function h and the Jacobian matrix $H_{k}$ can be described, respectively, as:

(24) $$h={\left[v_{x},v_{y},v_{z},a_{x},a_{y},a_{z},P_{JB}\right]}^{T}$$

and

(25) $$H_{k}\overset{\Delta }{=}\frac{\partial h}{\partial x_{k}}{|}_{\left({\hat{x}}_{J,k-1},{\hat{y}}_{J,k-1},{\hat{z}}_{J,k-1}\right)}={\left[\begin{array}{ccccccccc} 0 & 0 & 0 & 1 & 0 & 0 & 0 & 0 & 0\\ 0 & 0 & 0 & 0 & 1 & 0 & 0 & 0 & 0\\ 0 & 0 & 0 & 0 & 0 & 1 & 0 & 0 & 0\\ 0 & 0 & 0 & 0 & 0 & 0 & 1 & 0 & 0\\ 0 & 0 & 0 & 0 & 0 & 0 & 0 & 1 & 0\\ 0 & 0 & 0 & 0 & 0 & 0 & 0 & 0 & 1\\ \frac{\partial P_{JB}}{\partial x_{J}} & \frac{\partial P_{JB}}{\partial y_{J}} & \frac{\partial P_{JB}}{\partial z_{J}} & 0 & 0 & 0 & 0 & 0 & 0 \end{array}\right]}_{\left({\hat{x}}_{J,k-1},{\hat{y}}_{J,k-1},{\hat{z}}_{J,k-1}\right)}$$

In order to evaluate the partial derivatives appearing in the above Jacobian matrix, we have to utilize the relation between the received power ($P_{JB}$) and distance of jammer from the boundary node ($d_{JB}$) given in Equation (1). The distance of jammer from the boundary node can be evaluated while using the following expression:

(26) $$d_{JB}=\sqrt{{\left(x_{B}-x_{J}\right)}^{2}+{\left(y_{B}-y_{J}\right)}^{2}+{\left(z_{B}-z_{J}\right)}^{2}}$$

where ($x_{B},y_{B},z_{B}$) is the boundary node. Thus, the first derivative of jamming power with respect to jammer’s position at time *k* becomes as follows:

(27) $$\frac{\partial P_{JB}}{\partial x_{J}}{|}_{{\hat{x}}_{J,k-1}}=C\frac{x_{B}{\hat{x}}_{J,k-1}}{{\left(x_{B}+{\hat{x}}_{J,k-1}\right)}^{2}+{\left(y_{B}+{\hat{y}}_{J,k-1}\right)}^{2}+{\left(z_{B}+{\hat{z}}_{J,k-1}\right)}^{2}}$$

(28) $$\frac{\partial P_{JB}}{\partial y_{J}}{|}_{{\hat{y}}_{J,k-1}}=C\frac{y_{B}{\hat{y}}_{J,k-1}}{{\left(x_{B}+{\hat{x}}_{J,k-1}\right)}^{2}+{\left(y_{B}+{\hat{y}}_{J,k-1}\right)}^{2}+{\left(z_{B}+{\hat{z}}_{J,k-1}\right)}^{2}}$$

(29) $$\frac{\partial P_{JB}}{\partial z_{J}}{|}_{{\hat{z}}_{J,k-1}}=C\frac{z_{B}{\hat{z}}_{J_{k-1}}}{{\left(x_{B}+{\hat{x}}_{J_{k-1}}\right)}^{2}+{\left(y_{B}+{\hat{y}}_{J_{k-1}}\right)}^{2}+{\left(z_{B}+{\hat{z}}_{J_{k-1}}\right)}^{2}}$$

where *C* is a constant given by:

(30) $$C=\frac{10n}{ln10}$$

Hence, the proposed DEKF for the three-dimensional (3D) localization can be implemented while using the Equations (13)–(17) with the aid of Equations (20), (25)–(30).

### 4.2. Distance Ratio Based Distributed Extended Kalman Filter (DEKF-DR) for the 3D Localization

In this section, we proposed a distributed EKF by utilizing an additional edge node *E* in addition to a single boundary node *B*, as shown in Figure 4. For this purpose, we utilize the concept of Distance Ratio (β) described in Section 3. Because both the distances $d_{JB}$ and $d_{JE}$ are unknown and the SNR at the edge node equal to the system threshold value $\left(SNR_{E}\approx \gamma \right)$, we can estimate the distance ratio by SNR and power relations as follows:

(31) $$\beta =10^{\left(\frac{\gamma \;-\;P_{NB}\;+P_{JB}\;}{10n}\right)}$$

where $P_{NB}$ is the power received at the unaffected node *N* from the boundary node *B*. Thus, once the β is evaluated while using above relation, we can utilize it in Equations (8) and (9). Moreover, the distances can be expressed in terms of β as

(32) $$d_{JB}=\sqrt{{\left(x_{B}-{\hat{x}}_{J}\right)}^{2}+{\left(y_{B}-{\hat{y}}_{J}\right)}^{2}+{\left(z_{B}-{\hat{z}}_{J}\right)}^{2}}$$

(33) $$d_{JE}=d_{JB}\;\beta =\sqrt{{\left(x_{B}-{\hat{x}}_{J}\right)}^{2}+{\left(y_{B}-{\hat{y}}_{J}\right)}^{2}+{\left(z_{B}-{\hat{z}}_{J}\right)}^{2}}\;\left(\beta \right)$$

(34) $$d_{EB}=d_{JB}\;\left(1-\beta \right)=\sqrt{{\left(x_{B}-{\hat{x}}_{J}\right)}^{2}+{\left(y_{B}-{\hat{y}}_{J}\right)}^{2}+{\left(z_{B}-{\hat{z}}_{J}\right)}^{2}}\;\left(1-\beta \right)$$

Again, the state vector $x_{k}$, $A_{k}$ the Jacobian matrix, and the process noise $Q_{k}$ can be defined by Equations (18), (20) and (21), respectively. However, the measurement vector incorporates the power measurements from a single boundary node (i.e., $P_{JB}$) and from Edge node (i.e., $P_{JE}$ and $P_{EB}$), that is,

(35) $$h={\left[v_{x},v_{y},v_{z},a_{x},a_{y},a_{z},P_{JB},P_{JE},P_{EB}\right]}^{T}$$

therefore, the covariance matrix of measurement noise $R_{k}$ become

(36) $$R_{k}=diag\left(\sigma_{v_{x}}^{2},\sigma_{v_{y}}^{2},\sigma_{v_{z}}^{2},\sigma_{a_{x}}^{2},\sigma_{a_{y}}^{2},\sigma_{a_{z}}^{2},\sigma_{P_{JB}}^{2},\sigma_{P_{JE}}^{2},\sigma_{P_{EB}}^{2}\right)$$

Thus, the Jacobian matrix H can be obtained, as follows:

(37) $$H_{k}\overset{\Delta }{=}\frac{\partial h}{\partial x_{k}}{|}_{\left({\hat{x}}_{J,k-1},{\hat{y}}_{J,k-1},{\hat{z}}_{J,k-1}\right)}={\left[\begin{array}{ccccccccc} 0 & 0 & 0 & 1 & 0 & 0 & 0 & 0 & 0\\ 0 & 0 & 0 & 0 & 1 & 0 & 0 & 0 & 0\\ 0 & 0 & 0 & 0 & 0 & 1 & 0 & 0 & 0\\ 0 & 0 & 0 & 0 & 0 & 0 & 1 & 0 & 0\\ 0 & 0 & 0 & 0 & 0 & 0 & 0 & 1 & 0\\ 0 & 0 & 0 & 0 & 0 & 0 & 0 & 0 & 1\\ \frac{\partial P_{JB}}{\partial x} & \frac{\partial P_{JB}}{\partial y} & \frac{\partial P_{JB}}{\partial z} & 0 & 0 & 0 & 0 & 0 & 0\\ \frac{\partial P_{JE}}{\partial x} & \frac{\partial P_{JE}}{\partial y} & \frac{\partial P_{JE}}{\partial z} & 0 & 0 & 0 & 0 & 0 & 0\\ \frac{\partial P_{EB}}{\partial x} & \frac{\partial P_{EB}}{\partial y} & \frac{\partial P_{EB}}{\partial z} & 0 & 0 & 0 & 0 & 0 & 0 \end{array}\right]}_{\left({\hat{x}}_{J,k-1},{\hat{y}}_{J,k-1},{\hat{z}}_{J,k-1}\right)}$$

Therefore, the first derivative of jamming power with respect to jammer’s position at time *k* will be resulted in the following expressions:

(38) $$\frac{\partial P_{JB}}{\partial x_{J}}=C_{JB}\frac{x_{B}{\hat{x}}_{J_{k-1}}}{{\left(x_{B}+{\hat{x}}_{J_{k-1}}\right)}^{2}+{\left(y_{B}+{\hat{y}}_{J_{k-1}}\right)}^{2}+{\left(z_{B}+{\hat{z}}_{J_{k-1}}\right)}^{2}}$$

(39) $$\frac{\partial P_{JE}}{\partial x_{J}}=\frac{\partial P_{JB}}{\partial x_{J}}\;\beta_{k-1}$$

(40) $$\frac{\partial P_{EB}}{\partial x_{J}}=\frac{\partial P_{JB}}{\partial x_{J}}\;\left(1-\beta_{k-1}\right)$$

(41) $$\frac{\partial P_{JB}}{\partial y_{J}}=C_{JB}\frac{y_{B}{\hat{y}}_{J_{k-1}}}{{\left(x_{B}+{\hat{x}}_{J_{k-1}}\right)}^{2}+{\left(y_{B}+{\hat{y}}_{J_{k-1}}\right)}^{2}+{\left(z_{B}+{\hat{z}}_{J_{k-1}}\right)}^{2}}$$

(42) $$\frac{\partial P_{JE}}{\partial y_{J}}=\frac{\partial P_{JB}}{\partial y_{J}}\;\beta_{k-1}$$

(43) $$\frac{\partial P_{EB}}{\partial y_{J}}=\frac{\partial P_{JB}}{\partial y_{J}}\;\left(1-\beta_{k-1}\right)$$

(44) $$\frac{\partial P_{JB}}{\partial z}=C_{JB}\frac{z_{B}{\hat{z}}_{J_{k-1}}}{{\left(x_{B}+{\hat{x}}_{J_{k-1}}\right)}^{2}+{\left(y_{B}+{\hat{y}}_{J_{k-1}}\right)}^{2}+{\left(z_{B}+{\hat{z}}_{J_{k-1}}\right)}^{2}}$$

(45) $$\frac{\partial P_{JE}}{\partial z_{J}}=\frac{\partial P_{JB}}{\partial z_{J}}\;\beta_{k-1}$$

(46) $$\frac{\partial P_{EB}}{\partial z_{J}}=\frac{\partial P_{JB}}{\partial z_{J}}\;\left(1-\beta_{k-1}\right)$$

where,

(47) $$C_{JB}=\frac{10n}{ln10}$$

Thus, the proposed DEKF-DR algorithm for the 3D localization as explained in Algorithm 1 can be implemented while using the Equations (13)–(17) with the aid of Equations (37)–(47).
**Algorithm 1:**Pseudo-code of DEKF-DR1:Set the system threshold value = γ.2:Initialize time index (k) the boundary node index (i) and the neighbor node index (n).3:**Input:**$x_{k-1}$, $P_{k|k-1}z_{k}$.4:**Output:**$x_{k}$.5:**repeat**6:     k=k+1.7:     Capture the $P_{JB_{k}}$.8:     Estimate $P_{NB}$.9:     Compute the $\beta_{k}$ using Equation (5).10:    Compute the $P_{JE_{k}}$ and $P_{EB_{k}}$ using Equations (8) and (9).11:    ${\hat{x}}_{k|k-1}\leftarrow f\left({\hat{x}}_{k-1|k-1}\right)$.12:    $P_{k|k-1}\leftarrow A_{k}P_{k-1|k-1}A_{k}^{T}+Q_{k}$.13:    $z_{k}\leftarrow h\left(x_{k}\right)+v_{k}$.14:    Estimate $d_{JB_{k}}$ using Equation (1).15:    $d_{JE_{k}}\leftarrow \beta_{k}\;d_{JB_{k}}$.16:    $d_{EB_{k}}\leftarrow \left(1-\beta_{k}\right)\;d_{JB_{k}}$.17:    $K_{k}\leftarrow P_{k|k-1}H_{k}^{T}{\left(H_{k}P_{k|k-1}H_{k}^{T}+R_{k}\right)}^{-1}$.18:    ${\hat{x}}_{k}\leftarrow {\hat{x}}_{k|k-1}+K_{k}\left(z_{k}-h\left({\hat{x}}_{k|k-1}\right)\right)$.19:    $P_{k}\leftarrow P_{k|k-1}-K_{k}H_{k}P_{k|k-1}$20:**until** {Final time $=k_{f}$}

## 5. Extension of the Centralized Extended Kalman Filter (EKF-Centr) for the 3-D Localization

In the centralized EKF, every node near the jamming region may receive the jamming signal and transfer the collected data to a centralized base station, where the EKF is employed. Therefore, the EKF-Centr is designed to receive the jamming information at each time step *k* from all of the boundary nodes (say total $B_{i}$ boundary nodes). Thus, the state vector $x_{k}$ and $A_{k}$ the Jacobian matrix remains the same as appeared in Equations (18) and (20), respectively. However, the measurement vector incorporates the power measurements from all $B_{i}$ boundary nodes, which is,

(48) $$h={\left[v_{x},v_{y},v_{z},a_{x},a_{y},a_{z},P_{JB_{i}}^{1},P_{JB_{i}}^{2},\cdots ,P_{JB_{i}}^{B_{i}}\right]}^{T}$$

Additionally, the covariance matrix of measurement noise $R_{k}$

(49) $$R_{k}=diag\left(\sigma_{v_{x}}^{2},\sigma_{v_{y}}^{2},\sigma_{v_{z}}^{2},\sigma_{a_{x}}^{2},\sigma_{a_{y}}^{2},\sigma_{a_{z}}^{2},\sigma_{P_{JB_{i}}^{1}}^{2},\cdots ,\sigma_{P_{JB_{i}}^{B_{i}}}^{2}\right)$$

Consequently, the Jacobian matrix for the measurement will become:

(50) $$H_{k}\overset{\Delta }{=}\frac{\partial h}{\partial x_{k}}{|}_{\left({\hat{x}}_{J,k-1},{\hat{y}}_{J,k-1},{\hat{z}}_{J,k-1}\right)}={\left[\begin{array}{ccccccccc} 0 & 0 & 0 & 1 & 0 & 0 & 0 & 0 & 0\\ 0 & 0 & 0 & 0 & 1 & 0 & 0 & 0 & 0\\ 0 & 0 & 0 & 0 & 0 & 1 & 0 & 0 & 0\\ 0 & 0 & 0 & 0 & 0 & 0 & 1 & 0 & 0\\ 0 & 0 & 0 & 0 & 0 & 0 & 0 & 1 & 0\\ 0 & 0 & 0 & 0 & 0 & 0 & 0 & 0 & 1\\ \frac{\partial P_{JB_{i}}^{1}}{\partial x_{J}} & \frac{\partial P_{JB_{i}}^{1}}{\partial y_{J}} & \frac{\partial P_{JBi}^{1}}{\partial z_{J}} & 0 & 0 & 0 & 0 & 0 & 0\\ \frac{\partial P_{JB_{i}}^{2}}{\partial x_{J}} & \frac{\partial P_{JB_{i}}^{2}}{\partial y_{J}} & \frac{\partial P_{JB_{i}}^{2}}{\partial z_{J}} & 0 & 0 & 0 & 0 & 0 & 0\\ \; & \; & & \; & \vdots & \; & & \; & \\ \frac{\partial P_{JB_{i}}^{B_{i}}}{\partial x_{J}} & \frac{\partial P_{JB_{i}}^{B_{i}}}{\partial y_{J}} & \frac{\partial P_{JB_{i}}^{B_{i}}}{\partial z_{J}} & 0 & 0 & 0 & 0 & 0 & 0 \end{array}\right]}_{\left({\hat{x}}_{J,k-1},{\hat{y}}_{J,k-1},{\hat{z}}_{J,k-1}\right)}$$

Similar to the previous case, in order to evaluate the partial derivatives in the above, we utilize the distance of jammer from each boundary node. Thus, for the *i*-th boundary node, the jammer’s distance is given by:

(51) $$d_{JB}^{i}=\sqrt{{\left(x_{B}^{i}-x_{J}\right)}^{2}+{\left(y_{B}^{i}-y_{J}\right)}^{2}+{\left(z_{B}^{i}-z_{J}\right)}^{2}}$$

Therefore, the first derivative of jamming power with respect to jammer’s position will become:

(52) $$\frac{\partial P_{JB}^{i}}{\partial x_{J}}{|}_{{\hat{x}}_{J,k-1}}=C_{JB}^{i}\frac{x_{B}^{i}{\hat{x}}_{J,k-1}}{{\left(x_{B}^{i}+{\hat{x}}_{J,k-1}\right)}^{2}+{\left(y_{B}^{i}+{\hat{y}}_{J,k-1}\right)}^{2}+{\left(z_{B}^{i}+{\hat{z}}_{J,k-1}\right)}^{2}}$$

(53) $$\frac{\partial P_{JB}^{i}}{\partial y_{J}}{|}_{{\hat{y}}_{J,k-1}}=C_{JB}^{i}\frac{y_{B}^{i}{\hat{y}}_{J,k-1}}{{\left(x_{B}^{i}+{\hat{x}}_{J,k-1}\right)}^{2}+{\left(y_{B}^{i}+{\hat{y}}_{J,k-1}\right)}^{2}+{\left(z_{B}^{i}+{\hat{z}}_{J,k-1}\right)}^{2}}$$

(54) $$\frac{\partial P_{JB}^{i}}{\partial z_{J}}{|}_{{\hat{z}}_{J,k-1}}=C_{JB}^{i}\frac{z_{B}^{i}{\hat{z}}_{J,k-1}}{{\left(x_{B}^{i}+{\hat{x}}_{J,k-1}\right)}^{2}+{\left(y_{B}^{i}+{\hat{y}}_{J,k-1}\right)}^{2}+{\left(z_{B}^{i}+{\hat{z}}_{J,k-1}\right)}^{2}}$$

where $C_{JB}^{i}$ is the constant for the *i*-th node and is given by:

(55) $$C_{JB}^{i}=\frac{10n}{ln10}$$

In summary, the proposed EKF-Centr algorithm for the 3D localization can be implemented using the Equations (13)–(17) with the aid of Equations (50), (52)–(55).

**Remark** **1.** *At this stage, it is important to contrast the major differences among the two proposed algorithms (the DEKF and the DEKF-DR) and the conventional EKF-Centre. By observing the Jacobian matrix H, it can be deduced that the size of H is adjusted, depending on the number of nodes utilized in the localization process. In the DEKF, there is only one boundary node to detect the jammer’s location with the aid of received power only in distributed mechanism. In the EKF-Centr, there are $B_{i}$ nodes utilized to locate the jammer’s location by sending all of the information to a centralized node. Finally, in the DEKF-DR, a single boundary node along with an edge node is used in distributed fashion.*

## 6. Results and Discussion

We considered the first scenario in which the jammer UAV hovers in three-dimensional space (x,y,z) with constant acceleration equal to zero and variable velocity at each time step in order to evaluate the performance of the proposed algorithm. The boundary node or the tracker is located at the position (15,30,0) with transmitting power −35.5 dBm. The neighbor node near the boundary is located at (10,30,0) with the same transmitting power as that of the boundary node. The jammer UAV starts at position (10,20,12) with starting time $t_{0}$ equal to zero and transmitting power equals to −20 dBm. The sensing range of the boundary node is equal to 16 meters and the jammer UAV transmitting range is around 90 m. In the first experiment, the initial position for the original trajectory and UAV are (10,20,12).

**Figure 5:**
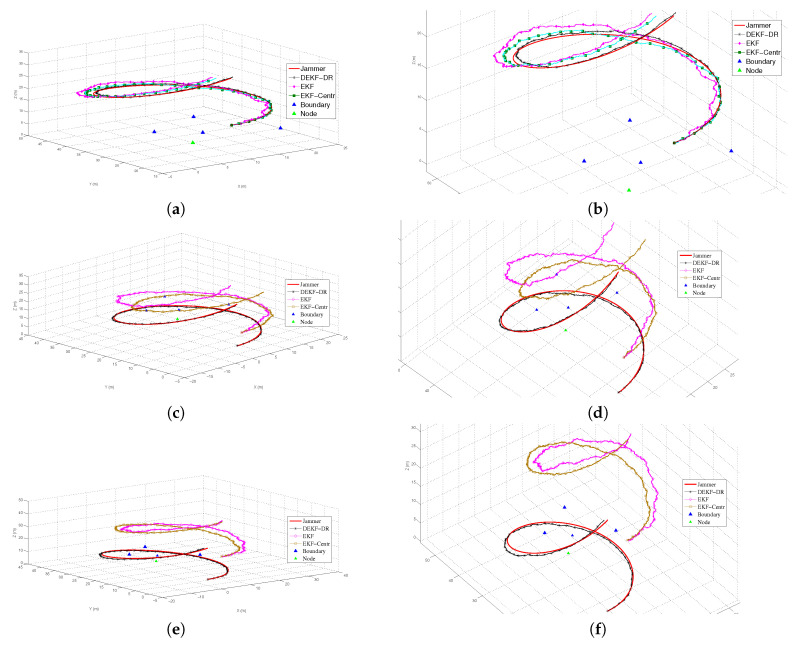
The UAV localization based on distance ratio (β) and Distributed Extended Kalman Filter (DEKF-DR) when compared to Centralized Extended Kalman Filter (EKF-Centr) and DEKF where the jammer UAV hovers over the target area to jam the base stations on the ground. (**a**) trajectory and UAV initial position (10,20,12), (**b**) Zoom in of (**a**). (**c**) trajectory starting position (0,0,5) and UAV initial position (5,4,8), (**d**) zoom in (**b**). (**e**) trajectory starting position (0,0,5) and UAV initial position (13,9,14), and (**f**) zoom in of (**e**).

It can be depicted with the results that are shown in Figure 5a that the proposed the DEKF-DR has better localization performance in comparison with the DEKF and the EKF-Centr. To measure the performance and the robustness of the proposed algorithm, we have set the starting positions different for both the original trajectory and the UAV, such that the original trajectory started at position (0,0,5), while the initial positions for the UAV are (5,4,8) in Figure 5c and (13,9,14) Figure 5e. It can be easily seen that the proposed DEKF-DR has outperformed its counterparts in both experiments and able to estimate the jammer more accurately. In contrast, the DEKF and EKF-Centr had a shift in tracking the jammer’s location, as shown in Figure 5c,e. Here, the blue triangle and the green triangle represent the boundary nodes and the neighbor nodes, respectively. The second scenario is to evaluate the centralized EKF, where the four boundary nodes located at positions (10,15,0), (5,25,0), (15,5,0) and (20,30,0), as shown in Figure 5. The system threshold value γ and the path loss factor *n* for free space set to 2, as in Table 2.

**Figure 6:**
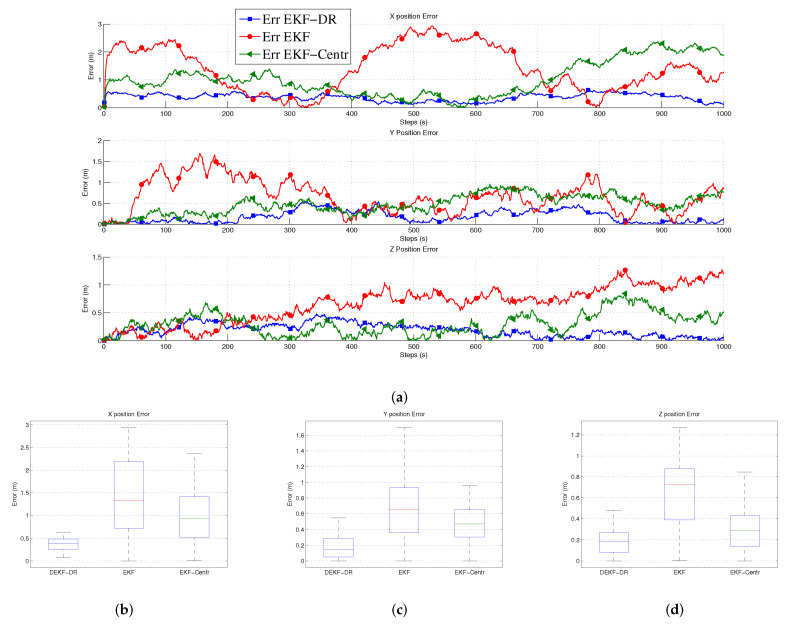
Representation of position error. (**a**) express the location errors at each time step on x, y, and z coordinates. the median error for three coordinates represented by the box plot in (**b**–**d**).

**Figure 7:**
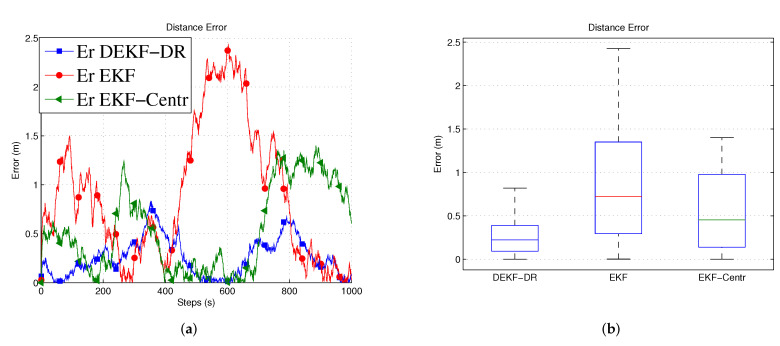
Distance Error. (**a**) Euclidean distance error; (**b**) lowest distance error.

**Figure 8:**
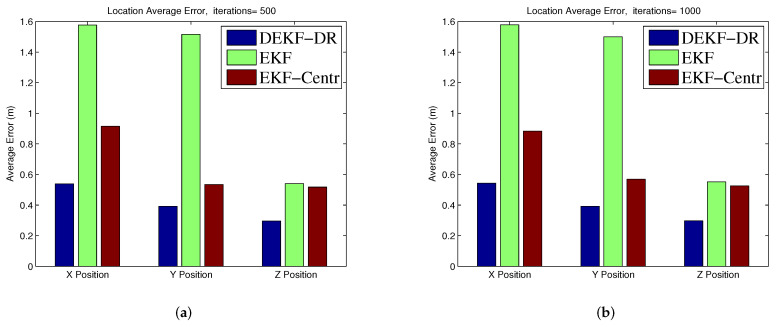
Average Error (**a**) 500 iterations and (**b**) 1000 iterations.

**Figure 9:**
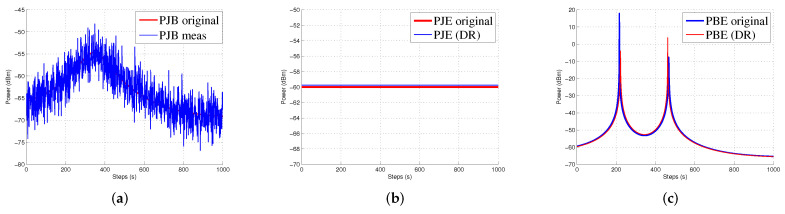
(**a**) Jamming power received by boundary node, (**b**) Jamming power estimated at the edge node and (**c**) jamming power estimated at the boundary node from the edge.

Figure 6a shows the position errors in three-dimension space (x, y, z) at each time step. Here, the jammer UAV is moving in 1000 steps to measure the performance of the EKF-DR. The proposed algorithm can estimate the jammer position more accurately when compared to the EKF-Centr and the DEKF. The maximum position error is less than 0.6 meters compared to 2.9 m and 2.4 m for the DEKF and the EKF-Centr, respectively. The DEKF-DR performed better as the average localization error is about 0.3 m on the x-axis, 0.1 m on the y-axis, and 0.18 on the z-axis, as shown in Figure 6b–d, respectively. In the same figure, the box plot represents the median of the localization error. Here, the median error for x position is less than 0.2 m compared to 1.3 m and 0.92 m for DEKF and EKF-Centr, respectively. Similarly, the median error for the z position is less than 0.29 m for the DEKF-DR compared to its counterparts. These results further verifies the superiority of the DEKF-DR to detect the jammer location in three-dimensional space. It can be seen from these results that the conventional EKF has reached sometime a very low error close to zero. However, its error is highly fluctuating and sometimes reaches to very high value. This is also illustrated in the statistical information provided by the box plot. These results show highly inconsistent behavior of estimation by the EKF, which is not desirable in real practice. On the other hand, the proposed EKF-DR has consistent estimation performance with lesser median localization error. Moreover, the DEKF-DR has lesser computational complexity than its counterparts. Furthermore, Figure 7a shows that the Euclidean distance error to the jammer. Here, it can be observed that the DEKF has poor performance, among others. This is due to the fact that it only uses one node information, which is insufficient for the Kalman filter to estimate the unknown position. On the other hand, the EKF-Centr is designed to receive the localization information from all four different nodes and, hence, it performed better than the DEKF. Finally, the DEKF-DR showed lowest distance error. as illustrated in Figure 7b. Its median error is about 0.3 m when compared to 0.7 m and 0.4 m for the DEKF and the EKF-Centr, respectively. In order to measure the performance of the proposed method, we run the simulation 500 times and 1000 times, as reported in Figure 8a,b, respectively. The average error of the DEKF-DR is around 0.56 m. We estimated the jamming power at different points using the distance ratio as shown in Figure 9. The jamming signal received by boundary node presented in Figure 9a and jamming signal at the edge node and from the edge to the boundary node presented in Figure 9b,c, respectively.

## 7. Conclusions

In this paper, we proposed two distributed EKF techniques for the 3-D UAV Jammer localization: the DEKF and the DEKF-DR. Moreover, the existing centralized EKF (EKF-Center) based method is also extended for the 3-D localization scenario. The DEKF and EKF-Center are only based on received power from the boundary nodes. However, the DEKF-DR utilized an additional edge node received power to formulate the problem in terms of Distance Ratio. Specifically, the DEKF-DR is based on the distance ratio estimated at the boundary node to estimate the distance between the jammer and the boundary node. The performance of the proposed distributed techniques was compared with the conventional centralized EKF based solution. Unlike the performance of the EKF-Centr, which depends on the number of cooperative nodes, the performance of the proposed DEKF and DEKF-DR are insensitive to the number of nodes, as they only rely on a single node. Moreover, the localization accuracy of the DEKF-DR is found to be the highest and it is not affected by the jammer location or its transmission power. Last but not the least, the DEKF-DR has a lesser computational load in contrast to the EKF-Center due to its distributed design.

## Figures and Tables

**Table 1:** List of symbols.

Notation	Description
*B*	Boundary node
$B_{i}$	Total boundary nodes
*N*	Unaffected node
$N_{E}$	Edge node
*J*	Jammer UAV
$N_{J}$	Jammed node
$(x_{J},y_{J},z_{J)}$	Jammer UAV position
$(x_{B},y_{B},z_{B)}$	Boundary node position
$(x_{N},y_{N},z_{N)}$	Node location
$p_{Jt}$	Jammer’s transmission power
$p_{t}$	Node’s transmission power
JRSS	Jammer received signal strength
RSS	Received signal strength
$P_{JB}^{i}$	Jamming power captured by boundary node i=1,2,⋯,n
$P_{JE}^{i}$	Jamming power estimated at edge node i=1,2,⋯,n
$P_{EB}^{i}$	Jamming power estimated at edge by boundary node i=1,2,⋯,n
$L_{i}$	Link quality for link i=1,2,⋯,n
SNR	Signal to noise ratio
γ	System threshold value
β	Distance ratio
$d_{JE}$	Distance from jammer to the edge node
$d_{EB}$	Distance from edge node to the boundary node
$d_{JB}$	Distance from jammer to the boundary node
*n*	Environmental factor
a	Acceleration
$x_{k}$	Current state
$x_{k-1}$	Previous state
$w_{k}$	Process noise
$z_{k}$	Measurement vector
$h_{k}$	Nonlinear function
$v_{k}$	Measurement noise
Q	Covariance matrix for prediction
R	Covariance matrix for observation
$H_{k}$	Jacobian matrix
$K_{k}$	Kalman gain
A	Transition matrix
v	Velocity
$X_{\sigma }$	Gaussian noise

**Table 2:** The Simulation Parameters.

Parameter	Meaning	Assigned Values
*L*	Range of network	100
*N*	Number of nodes	1
$N_{j}$	Number of jammer	1
$P_{Jt}$	Jammer’s transmission power (dBm)	−20 (dBm)
$P_{t}$	Sensor’s transmission Power (dBm)	−35.5 (dBm)
$R_{J}$	Jammer’s transmission range (m)	90 (m)
$R_{N}$	Node’s transmission range (m)	16 (m)
$k_{0}$	Initial time	0 s
$t_{}$	End time	1000 s
Δt	Time step	1.0 s
$v_{x}$	Initial velocity on x axis	1 m/s
$v_{y}$	Initial velocity on y axis	0 m/s
*a*	acceleration	0 m/s^2^
γ	System threshold value	2
$n_{}$	Environmental factor	2
